# Supplementary material for: Dissecting aggregation and seeding dynamics of α-Syn polymorphs using the phasor approach to FLIM
Source: Commun Biol. 2022 Dec 8;5:1345. doi: 10.1038/s42003-022-04289-6 (PMC9729209; doi:10.1038/s42003-022-04289-6)
Supplement: Supplementary file 2 — Supplementary Information [file 42003_2022_4289_MOESM2_ESM.pdf]

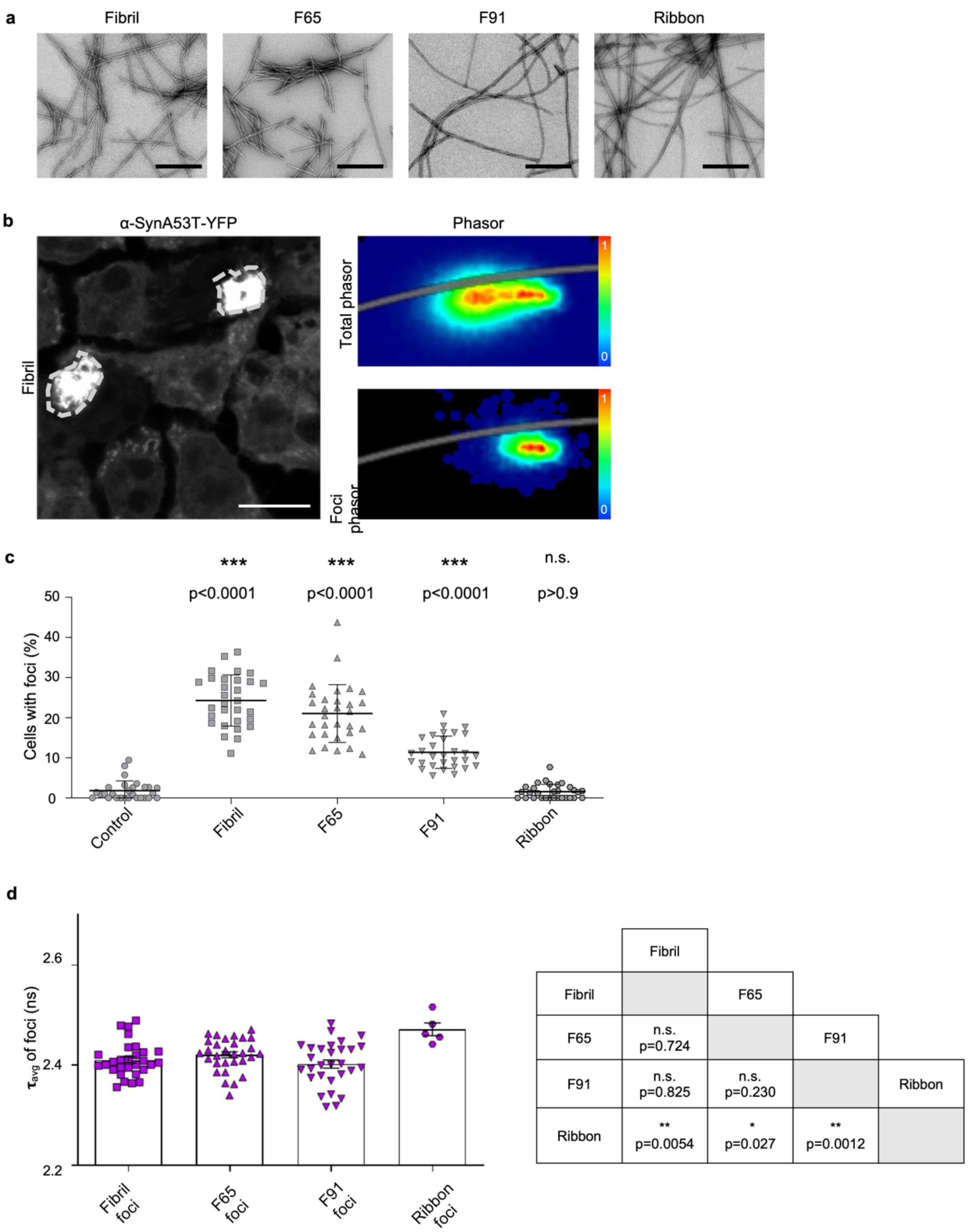

	Fibril	F65	F91	Ribbon
Fibril				
F65	n.s. p=0.724			
F91	n.s. p=0.825	n.s. p=0.230		
Ribbon	** p=0.0054	* p=0.027	** p=0.0012	

Supplementary Figure 1 Seeding of α-SynA53T-YFP biosensor cells with different unlabeled recombinant α-Syn fibrils. (**a**) Transmission electron microscopy (TEM) images of the different α-Syn polymorphs used throughout this study. Scale bar, 200 nm. (**b**) ROI drawn around foci in the fluorescence intensity image (dotted white line) induced by seeded aggregation show that foci contain α-SynA53T-YFP species with shorter lifetimes compared to the total phasor distribution. Scale bar, 10 µm. The phasor displays the relative occurrence of lifetimes in the accompanying image (0-1). (**c**) Quantification of cells with visible foci (30 images per condition). Statistical analysis was done using Kruskal-Wallis one-way analysis with Dunn's multiple comparison test. (**d**) The mean fluorescence lifetime (τ) of α-SynA53T-

YFP foci seeded with different α-Syn polymorphs. Data are shown as mean ± SEM. Statistical analysis was done using one-way ANOVA with Turkey's multiple comparison test.

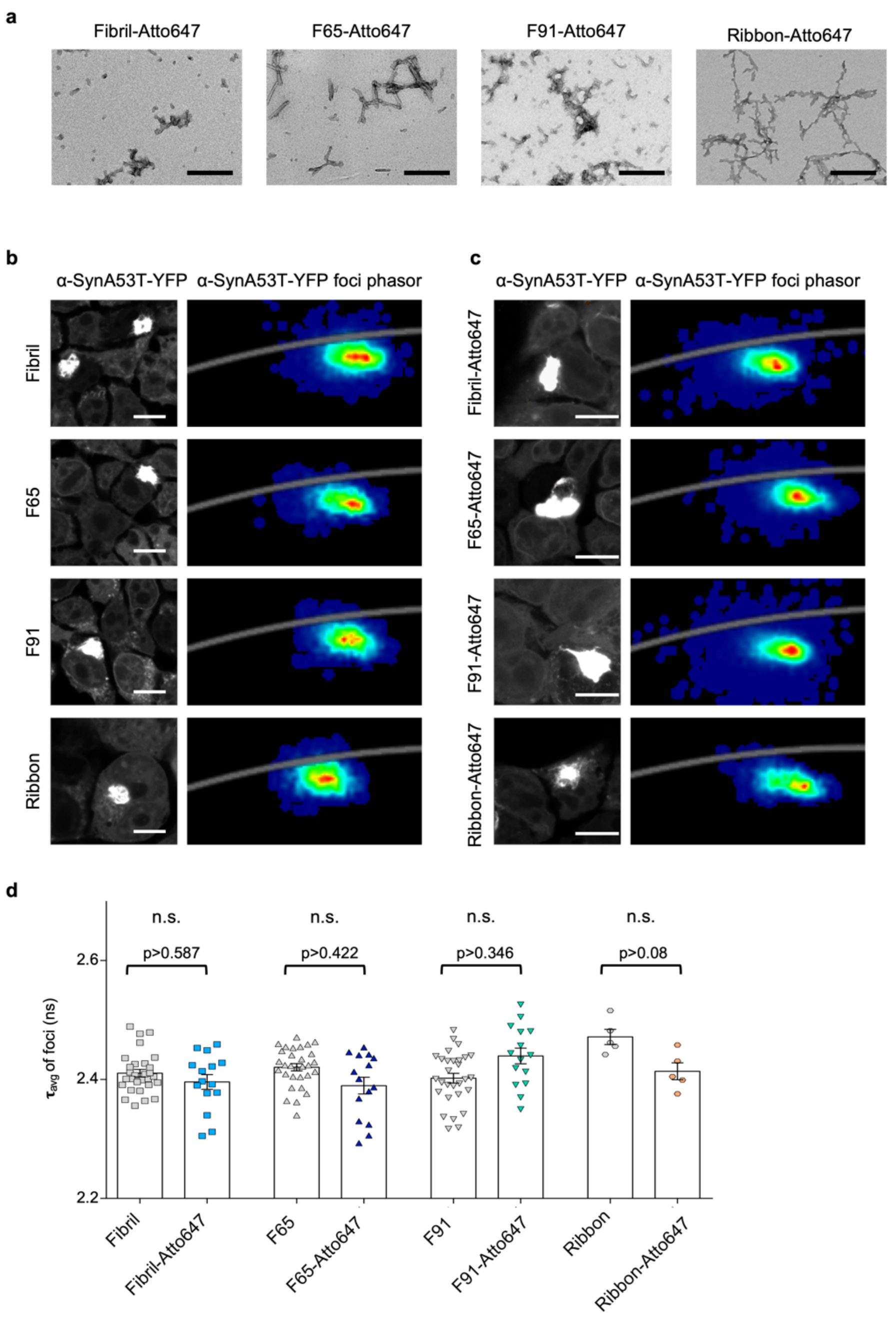

Supplementary Figure 2 FLIM analysis of α-SynA53T-YFP seeded with either unlabeled or Atto647-labeled α-Syn polymorphs. (**a**) Transmission electron microscopy (TEM) images of the different α-Syn polymorphs labeled with Atto647 used throughout this study. Scale bar, 200 nm. (**b**, **c**) Fluorescence intensity images of α-SynA53T-YFP seeded with either unlabeled or Atto647-labeled α-Syn polymorphs. Corresponding phasor plots show the fluorescence lifetimes of the foci in α-SynA53T-YFP with seeded aggregation. Scale bar, 10 μm. (**d**) The mean fluorescence lifetime (τ) of α-SynA53T-YFP foci seeded with different α-Syn polymorphs unlabeled or labeled with Atto647. Data are shown as mean ± SEM. Statistical analysis was done using multiple t-test correcting for multiple comparisons using the Holm-Sidak method.

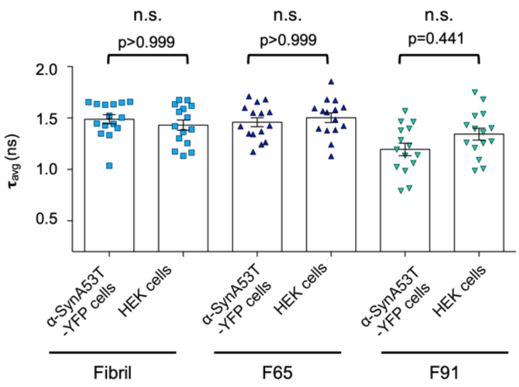

Supplementary Figure 3 Comparison of the mean fluorescence lifetime (t) of polymorphs in HEK cells and HEK cells expressing α-SynA53T-YFP. Data are shown as mean ± SEM. Statistical analysis was done using Kruskal-Wallis one-way analysis with Dunn's multiple comparison test.

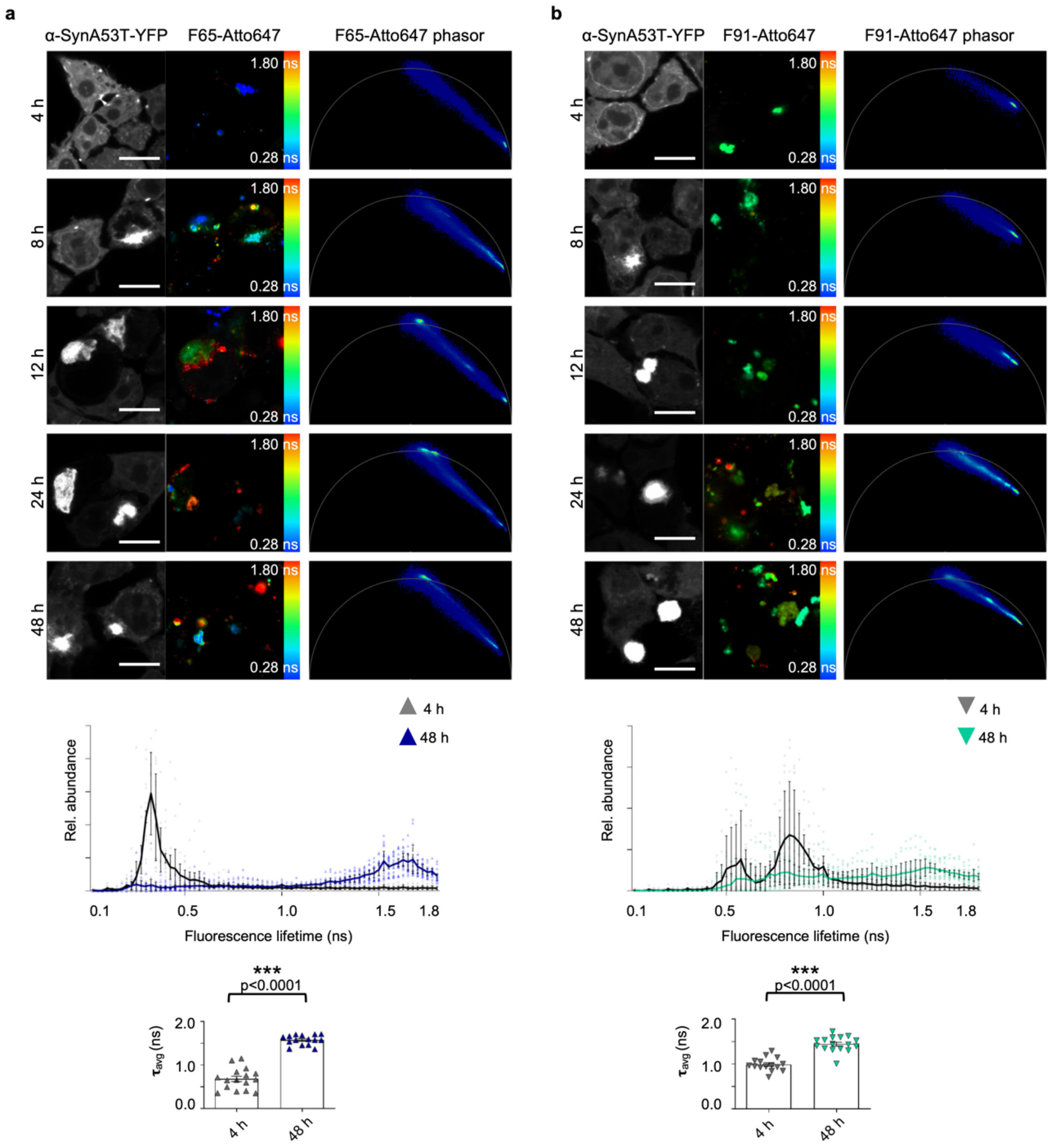

Supplementary Figure 4 Kinetics of F65 and F91 seed processing. (**a**, **b**) Fluorescence intensity images of α-SynA53T-YFP and indicated α-Syn polymorphs labeled with Atto647 pseudo colored from blue (0.28 ns) to red (1.8 ns) to illustrate short (blue) and long (red) fluorescence lifetimes with the merged images and the corresponding phasor plots of the fluorescence lifetime of seeds at indicated timepoints after seeding. Histograms of α-Syn-Atto647 seeds showing the relative abundance (rel. abundance) of pixels with a certain fluorescence lifetime (ranging from 0 to 1.8 ns), at 4h and 48h, averaged over all acquired images (n = 15 for each timepoint, mean + SEM). F65 and F91 seeds accumulate species with longer fluorescence lifetimes in a time dependent manner. Scale bar, 10 µm. Comparison of the mean fluorescence lifetime (τ) of F65 and F91 at 4 h and 48 h. Data are shown as mean ± SEM. Statistical analysis was done using unpaired t-test.

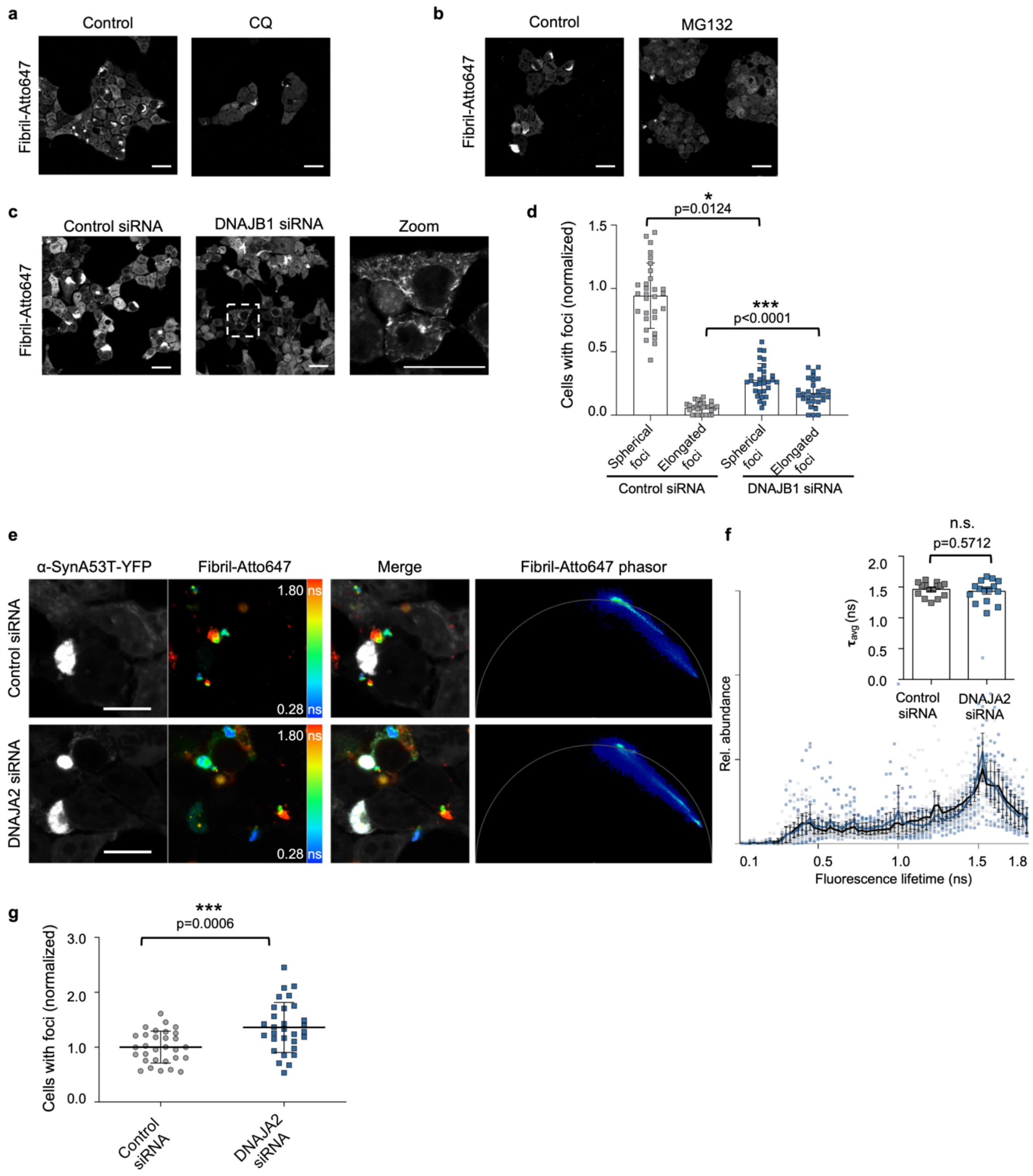

Supplementary Figure 5 Blocking the cellular processing of Fibril seeds reduces their seeding efficiency. (**a**, **b**) Representative fluorescence intensity images of α-SynA53T-YFP cells seeded with Fibril-Atto647 and treated with MG132 or chloroquine (CQ). Scale bar, 20 µm. (**c**) Representative image of α-SynA53T-YFP cells seeded with Fibril-Atto647 treated with control or DNAJB1 siRNA. Zoom shows elongated foci seen in cells seeded with Fibril-Atto647 and treated with DNAJB1 siRNA Scale bar, 20 µm. (**d**) Quantification of cells with visible elongated and spherical foci upon DNJAB1 siRNA. Statistical analysis was done using two-way ANOVA with Sidak's multiple comparison test. (**e**) Fluorescence intensity images of α-SynA53T-YFP and Fibril-Atto647 seeds pseudo colored from blue (0.28 ns) to red (1.8 ns)

to indicate short (blue) and long (red) fluorescence lifetimes with the merged images and the corresponding phasor plots of the fluorescence lifetime of Fibril-Atto647 seeds upon treatment with control or DNAJA2 siRNA. Scale bar, 10 µm. (**f**) Histograms of Fibril-Atto647 showing the relative abundance (rel. abundance) of pixels with a certain fluorescence lifetime, averaged over all acquired images (n = 15 for each condition). The mean fluorescence lifetime (τ) of Fibril-Atto647 upon treatment. Statistical analysis was done using unpaired t-test. n.s. = not significant. (**g**) Quantification of cells with visible foci after seeding with Fibril-Atto647. Statistical analysis was done using an unpaired t-test. Data are shown as mean ± SEM.

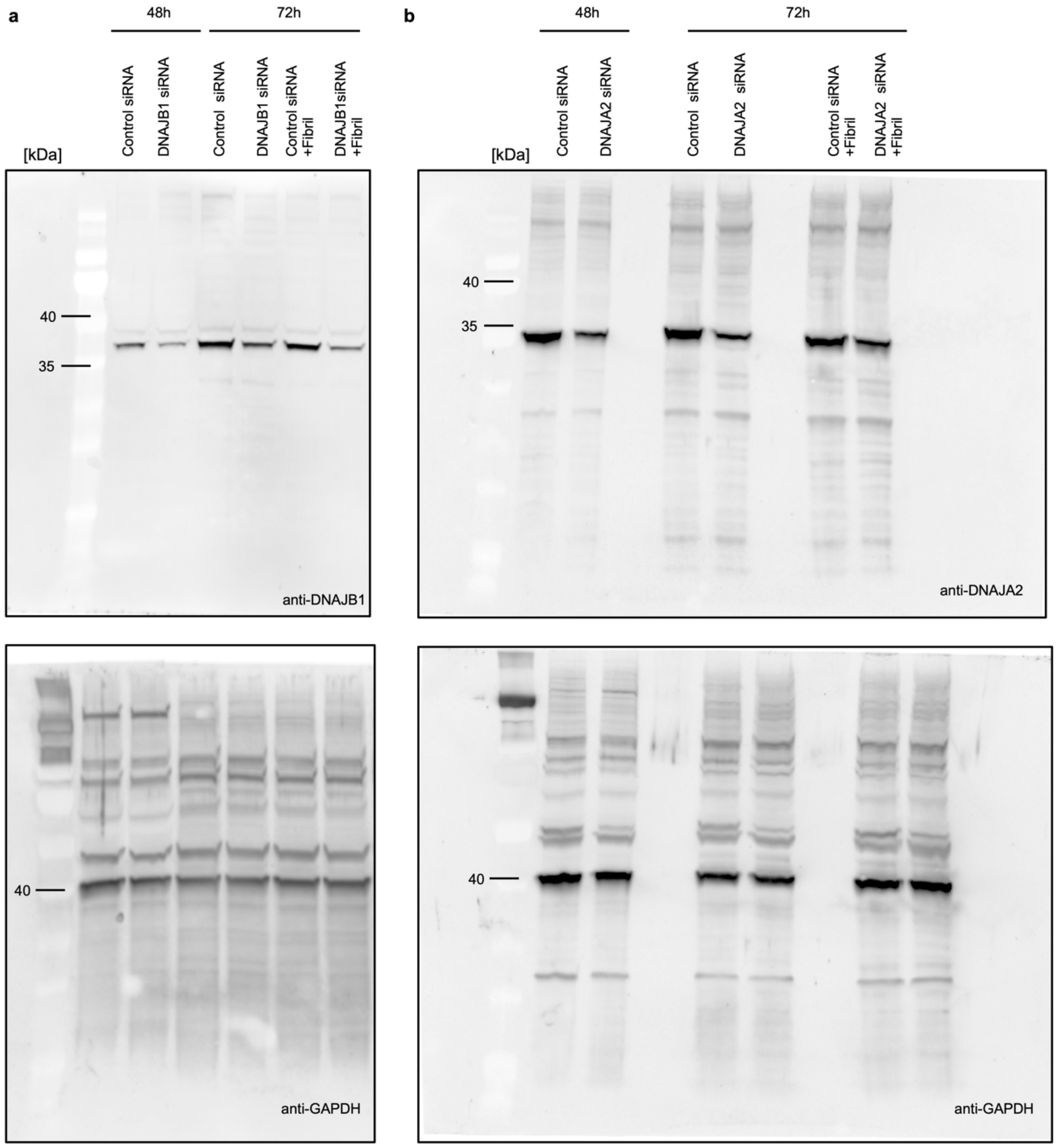

Supplementary Figure 6 Assessment of the knockdown efficiency of the DNAJB1 and DNAJA2 siRNAas. (**a**) Uncropped western blot to detect DNAJB1 levels when Fibril-Alex647 are added (48h) and after seeding for 24h (72h). Anti-GAPDH was used as a loading control. (**b**) Uncropped western blot to detect DNAJA2 levels when Fibril-Atto647 seeds are added (48h) and after seeding for 24h (72h). Anti-GAPDH was used as a loading control.

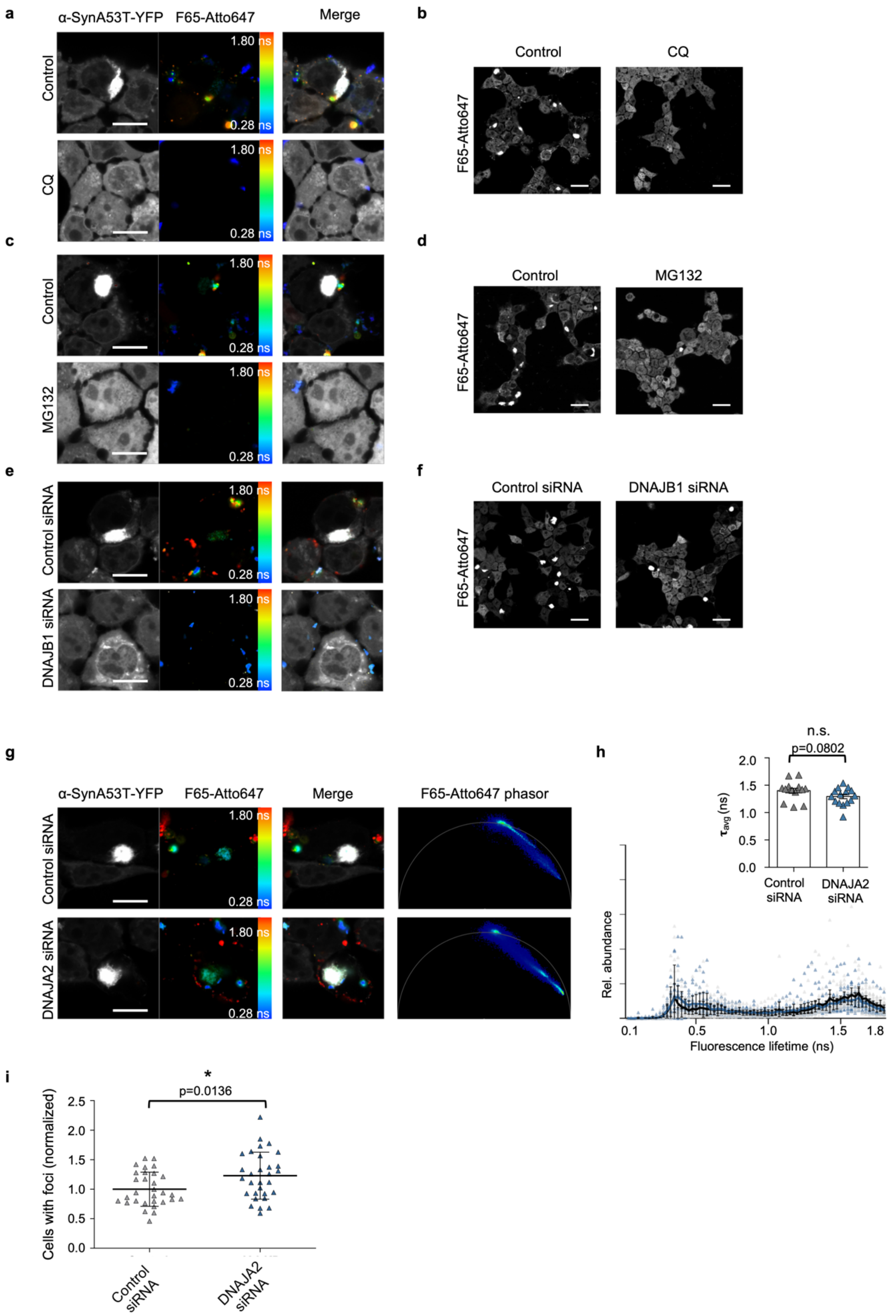

Supplementary Figure 7 Blocking the cellular processing of F65 seeds reduces their seeding efficiency. (**a**, **c**, **e**) Fluorescence intensity images of α-SynA53T-YFP and F65-Atto647 seeds pseudo colored from blue (0.28 ns) to red (1.8 ns) to illustrate short (blue) and

long (red) fluorescence lifetimes with the merged images of the fluorescence lifetime of F65-Atto647 seeds upon treatment with chloroquine (CQ) (**a**), MG132 (**c**), or DNAJB1 siRNA (**e**), which correspond to the phasor plots in Figure 5. Scale bar, 10 μm. (b, d, f) Representative fluorescence intensity images of α-SynA53T-YFP cells seeded with F65-Atto647 and treated with chloroquine (CQ) (**b**), MG132 (**d**) or DNAJB1 siRNA (**f**). Scale bar, 20 μm. (**g**) Fluorescence intensity images of α-SynA53T-YFP and F65-Atto647 seeds pseudo colored and the corresponding phasor plots of the fluorescence lifetime of F65-Atto647 seeds upon treatment with control or DNAJA2 siRNA. Scale bar, 10 μm. (**h**) Histograms of F65-Atto647 showing the relative abundance (rel. abundance) of pixels with a certain fluorescence lifetime, averaged over all acquired images (n = 15 for each condition). The mean fluorescence lifetime (τ) of Fibril-Atto647 upon treatment. Statistical analysis was done using unpaired t-test. (**i**) Quantification of cells with visible foci after seeding with F65-Atto647. Statistical analysis was done using an unpaired t-test. Data are shown as mean ± SEM.

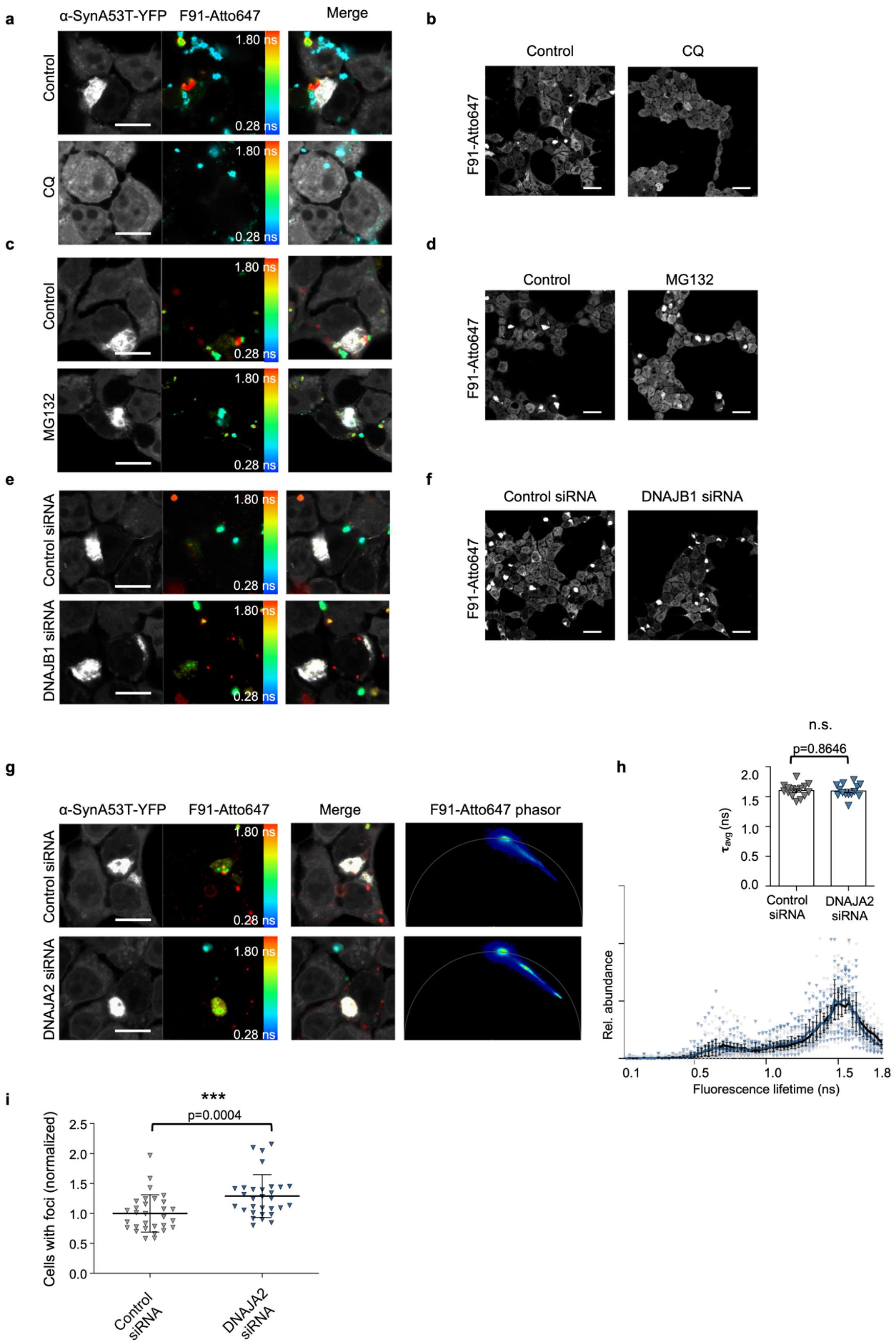

Supplementary Figure 8 Blocking the cellular processing of F91 seeds reduces their seeding efficiency. (**a**, **c**, **e**) Fluorescence intensity images of α-SynA53T-YFP and F91-Atto647 seeds pseudo colored from blue (0.28 ns) to red (1.8 ns) to illustrate short (blue) and

long (red) fluorescence lifetimes with the merged images of the fluorescence lifetime of F91-Atto647 seeds upon treatment with chloroquine (CQ) (**a**), MG132 (**c**), or DNAJB1 siRNA (**e**), which correspond to phasor plots in Figure 5. Scale bar, 10 μm. (**b**, **d**, **f**) Representative fluorescence intensity images of α-SynA53T-YFP cells seeded with F91-Atto647 and treated with chloroquine (CQ) (**b**), MG132 (**d**) or DNAJB1 siRNA (**f**). Scale bar, 20 μm. (**g**) Fluorescence intensity images of α-SynA53T-YFP and F91-Atto647 seeds pseudo colored and the corresponding phasor plots of the fluorescence lifetime of F91-Atto647 seeds upon treatment with control or DNAJA2 siRNA. Scale bar, 10 μm. (**h**) Histograms of F91-Atto647 showing the relative abundance (rel. abundance) of pixels with a certain fluorescence lifetime, averaged over all acquired images (n = 15 for each condition). The mean fluorescence lifetime (τ) of Fibril-Atto647 upon treatment. Statistical analysis was done using unpaired t-test. (**i**) Quantification of cells with visible foci after seeding with F91-Atto647. Statistical analysis was done using an unpaired t-test. Data are shown as mean ± SEM.
