# Supplementary material for: Dissecting aggregation and seeding dynamics of α-Syn polymorphs using the phasor approach to FLIM
Source: Commun Biol. 2022 Dec 8;5:1345. doi: 10.1038/s42003-022-04289-6 (PMC9729209; doi:10.1038/s42003-022-04289-6)
Supplement: Supplementary file 3 — Description of Additional Supplementary Files [file 42003_2022_4289_MOESM3_ESM.pdf]

# Description of Additional Supplementary Files

File name: Supplementary Data 1

Description: The source data for Figure 1

File name: Supplementary Data 2

Description: The source data for Figure 2

File name: Supplementary Data 3

Description: The source data for Figure 3

File name: Supplementary Data 4

Description: The source data for Figure 4

File name: Supplementary Data 5

Description: The source data for Figure 5

File name: Supplementary Data 6

Description: The source data for Figure 6

File name: Supplementary Data 7

Description: The source data for Supplementary Figure 1

File name: Supplementary Data 8

Description: The source data for Supplementary Figure 2

File name: Supplementary Data 9

Description: The source data for Supplementary Figure 3

File name: Supplementary Data 10

Description: The source data for Supplementary Figure 4

File name: Supplementary Data 11

Description: The source data for Supplementary Figure 5

File name: Supplementary Data 12

Description: The source data for Supplementary Figure 6

File name: Supplementary Data 13

Description: The source data for Supplementary Figure 7
