# Supplementary material for: Dissecting aggregation and seeding dynamics of α-Syn polymorphs using the phasor approach to FLIM
Source: Commun Biol. 2022 Dec 8;5:1345. doi: 10.1038/s42003-022-04289-6 (PMC9729209; doi:10.1038/s42003-022-04289-6)
Supplement: Supplementary file 17 — Reporting Summary [file 42003_2022_4289_MOESM17_ESM.pdf]

nature portfolio

Corresponding author(s): Carmen Nussbaum-Krammer

Last updated by author(s): Nov 10, 2022

Reporting Summary

Nature Portfolio wishes to improve the reproducibility of the work that we publish. This form provides structure for consistency and transparency in reporting. For further information on Nature Portfolio policies, see our Editorial Policies and the Editorial Policy Checklist.

Statistics

For all statistical analyses, confirm that the following items are present in the figure legend, table legend, main text, or Methods section.

n/a	Confirmed	
☐	☒	The exact sample size (*n*) for each experimental group/condition, given as a discrete number and unit of measurement
☐	☒	A statement on whether measurements were taken from distinct samples or whether the same sample was measured repeatedly
☐	☒	The statistical test(s) used AND whether they are one- or two-sided<br>*Only common tests should be described solely by name; describe more complex techniques in the Methods section.*
☐	☒	A description of all covariates tested
☐	☒	A description of any assumptions or corrections, such as tests of normality and adjustment for multiple comparisons
☐	☒	A full description of the statistical parameters including central tendency (e.g. means) or other basic estimates (e.g. regression coefficient) AND variation (e.g. standard deviation) or associated estimates of uncertainty (e.g. confidence intervals)
☐	☒	For null hypothesis testing, the test statistic (e.g. *F*, *t*, *r*) with confidence intervals, effect sizes, degrees of freedom and *P* value noted<br>*Give P values as exact values whenever suitable.*
☒	☐	For Bayesian analysis, information on the choice of priors and Markov chain Monte Carlo settings
☒	☐	For hierarchical and complex designs, identification of the appropriate level for tests and full reporting of outcomes
☒	☐	Estimates of effect sizes (e.g. Cohen's *d*, Pearson's *r*), indicating how they were calculated

Our web collection on statistics for biologists contains articles on many of the points above.

Software and code

Policy information about availability of computer code

Data collection	Leica Application Suite 3 (TCS SP8 STED 3X FALCON FLIM)
Data analysis	Graphpad prism 6. Fiji for Mac OS X

For manuscripts utilizing custom algorithms or software that are central to the research but not yet described in published literature, software must be made available to editors and reviewers. We strongly encourage code deposition in a community repository (e.g. GitHub). See the Nature Portfolio guidelines for submitting code & software for further information.

Data

Policy information about availability of data

All manuscripts must include a data availability statement. This statement should provide the following information, where applicable:

- Accession codes, unique identifiers, or web links for publicly available datasets
- A description of any restrictions on data availability
- For clinical datasets or third party data, please ensure that the statement adheres to our policy

All authors confirm that all relevant data are included in the paper and its supplementary information file. Additional files can be provided upon request.

Human research participants

Policy information about studies involving human research participants and Sex and Gender in Research.

Reporting on sex and gender	Not applicable
Population characteristics	Not applicable
Recruitment	Not applicable
Ethics oversight	Not applicable

Note that full information on the approval of the study protocol must also be provided in the manuscript.

Field-specific reporting

Please select the one below that is the best fit for your research. If you are not sure, read the appropriate sections before making your selection.

☒ Life sciences ☐ Behavioural & social sciences ☐ Ecological, evolutionary & environmental sciences

For a reference copy of the document with all sections, see nature.com/documents/nr-reporting-summary-flat.pdf

Life sciences study design

All studies must disclose on these points even when the disclosure is negative.

Sample size	No statistical methods were used to predetermine sample size. Standard sample size from the field were used.
Data exclusions	No data was excluded.
Replication	All experiments were repeated 3 times. FLIM analysis was done on random cells with foci. Microscopy images and western blots are representative of at least three individual experiments. All image quantifications are shown as the mean of replicates acquired in randomly chosen microscopy areas.
Randomization	Not relevant in our study as it only involves experiments with cell lines.
Blinding	No blinding was done but bias was impossible because only the FLIM analysis revealed the aggregate species composition in the cells, which is not visible before starting the FLIM analysis. No data was excluded.

Reporting for specific materials, systems and methods

We require information from authors about some types of materials, experimental systems and methods used in many studies. Here, indicate whether each material, system or method listed is relevant to your study. If you are not sure if a list item applies to your research, read the appropriate section before selecting a response.

Materials & experimental systems

n/a	Involved in the study	
☐	☒	Antibodies
☐	☒	Eukaryotic cell lines
☒	☐	Palaeontology and archaeology
☒	☐	Animals and other organisms
☒	☐	Clinical data
☒	☐	Dual use research of concern

Methods

n/a	Involved in the study	
☒	☐	ChIP-seq
☒	☐	Flow cytometry
☒	☐	MRI-based neuroimaging

Antibodies

Antibodies used	anti-HSP40/Hdj1 (2E1, Enzo), anti-DNAJA2 (ab147216, Abcam), Anti-GAPDH antibody (clone GAPDH-71.1, Sigma-Aldrich
Validation	These antibodies are commercial antibodies and have been previously used and validated. See individual information provided online on the company website.

Eukaryotic cell lines

Policy information about cell lines and Sex and Gender in Research

Cell line source(s)	EK293T α-SynA53T-YFP was kindly provided by Marc Diamond (MTA)<br>HEK293T
Authentication	The original HEK293T cells were received from ATCC with authentication certificate from supplier. The HEK293T α-SynA53T-YFP was kindly provided by Marc Diamond (MTA). α-SynA53T-YFP expression was validated in microscopy and western blot.
Mycoplasma contamination	Cell lines tested negative for mycoplasma contamination.
Commonly misidentified lines<br>(See ICLAC register)	Not applicable.
